# Residential Proximity to Methyl Bromide Use and Birth Outcomes in an Agricultural Population in California

**DOI:** 10.1289/ehp.1205682

**Published:** 2013-04-19

**Authors:** Alison Gemmill, Robert B. Gunier, Asa Bradman, Brenda Eskenazi, Kim G. Harley

**Affiliations:** 1 Department of Demography, University of California, Berkeley, Berkeley, California, USA; 2 Center for Environmental Research and Children’s Health, School of Public Health, University of California, Berkeley, Berkeley, California, USA

**Keywords:** birth outcomes, birth weight, fumigants, methyl bromide, pesticides, residential proximity

## Abstract

Background: Methyl bromide, a fungicide often used in strawberry cultivation, is of concern for residents who live near agricultural applications because of its toxicity and potential for drift. Little is known about the effects of methyl bromide exposure during pregnancy.

Objective: We investigated the relationship between residential proximity to methyl bromide use and birth outcomes.

Methods: Participants were from the CHAMACOS (Center for the Health Assessment of Mothers and Children of Salinas) study (*n* = 442), a longitudinal cohort study examining the health effects of environmental exposures on pregnant women and their children in an agricultural community in northern California. Using data from the California Pesticide Use Reporting system, we employed a geographic information system to estimate the amount of methyl bromide applied within 5 km of a woman’s residence during pregnancy. Multiple linear regression models were used to estimate associations between trimester-specific proximity to use and birth weight, length, head circumference, and gestational age.

Results: High methyl bromide use (vs. no use) within 5 km of the home during the second trimester was negatively associated with birth weight (β = –113.1 g; CI: –218.1, –8.1), birth length (β = –0.85 cm; CI: –1.44, –0.27), and head circumference (β = –0.33 cm; CI: –0.67, 0.01). These outcomes were also associated with moderate methyl bromide use during the second trimester. Negative associations with fetal growth parameters were stronger when larger (5 km and 8 km) versus smaller (1 km and 3 km) buffer zones were used to estimate exposure.

Conclusions: Residential proximity to methyl bromide use during the second trimester was associated with markers of restricted fetal growth in our study.

Methyl bromide is a fumigant used for pre-planting soil preparation. Because of concerns about its effect on the ozone layer, methyl bromide was banned in 2005 under the Montreal Protocol (United Nations Environment Programme 2000). Before the ban, methyl bromide was one of the most heavily used pesticides in California, with nearly 5 million kg applied in 2000 [California Department of Pesticide Regulation (DPR) 2001b]. Although methyl bromide use has declined in recent years, > 1.75 million kg was applied throughout the state in 2010 due to critical use exemptions for strawberries and other agricultural commodities (California DPR 2012c). Critical use of methyl bromide in the United States has declined markedly since 2005, but authorizations for use are still granted by the U.S. Environmental Protection Agency (U.S. EPA 2012).

Animal studies of methyl bromide exposure suggest the potential for developmental toxicity (National Research Council 2000). In an inhalation study of New Zealand white rabbits, exposure to 80 ppm methyl bromide during gestational days 7–19 resulted in decreased fetal body weights and increased rates of fetal malformations including gall bladder agenesis and fused sternebrae (National Research Council 2000). In another inhalation study, pups born to dams exposed to 20 and 70 ppm during gestation had increased rates of skull ossification defects (National Research Council 2000). A similar study of rats found dose-dependent reductions in fetal body and brain weights, as well as reduced cerebral cortex widths (National Research Council 2000). Based on this limited evidence, the California DPR lists methyl bromide as a probable developmental toxicant (National Research Council 2000).

Several human poisoning case studies indicate that high levels of acute methyl bromide exposure are associated with dermal burns, neurological impairment, and death (Breeman 2009; Langård et al. 1996; Lifshitz and Gavrilov 2000). Much less is known about the effects of chronic, low-level exposure in human populations (National Research Council 2000). In a sample of 56 male workers, long-term occupational exposure to methyl bromide was associated with chronic symptoms of dizziness, numbness, nightmares, and fatigue (Kishi et al. 1991). Other studies have investigated the health effects of residential proximity to methyl bromide use as part of larger analyses of ambient pesticide exposure. To date, one study reported evidence of an association with prostate cancer (Cockburn et al. 2011), but studies of breast cancer (Reynolds et al. 2004), childhood cancers (Reynolds et al. 2005), and autism spectrum disorders (Roberts et al. 2007) did not report associations with proximity to methyl bromide use.

Unlike most pesticides, fumigants such as methyl bromide have a high vapor pressure and are more likely to drift off-site (Woodrow and Krieger 2007). It is estimated that 30–50% of agricultural applications of methyl bromide are released into the air even when protective measures such as plastic tarps are in place (Honaganahalli and Seiber 2000; Majewski et al. 1995; Yagi et al. 1993). Because inhalation is the primary route of exposure to methyl bromide, ambient air concentrations are important for assessing exposure to nearby residents (National Research Council 2000). In agricultural areas of California, measured outdoor concentrations of methyl bromide averaged 0.1–7.7 ppb over an 8-week monitoring period in 2000 (California Air Resources Board 2001), or up to one-tenth of the levels associated with adverse outcomes in some animal studies (Breslin et al. 1990; Sikov et al. 1981). In addition, methyl bromide has been detected in air > 70 km away from the nearest application site (Honaganahalli and Seiber 2000).

Chronic exposure to methyl bromide is difficult to assess because there are no exposure biomarkers (Minnesota Department of Health 1999). The few studies that have assessed chronic methyl bromide exposure have done so by residential proxy or occupational history reports (Cockburn et al. 2011; Kishi et al. 1991; Reynolds et al. 2004, 2005; Roberts et al. 2007). Studies in California can use extensive information on the timing, location, and amount of agricultural methyl bromide applications, which must be reported to the state’s Pesticide Use Reporting (PUR) system (California DPR 2012b).

In the current study we examined whether residential proximity to methyl bromide applications is associated with fetal growth and gestational length in a cohort of pregnant women living in an agricultural community in the Salinas Valley of California. We linked California PUR data with birth outcome and demographic data for pregnant women participating in the Center for the Health Assessment of Mothers and Children of Salinas (CHAMACOS) study, a longitudinal cohort study examining the health effects of environmental exposures on pregnant women and their children living in an agricultural community in northern California.

## Methods

*Participants.* Women were recruited for the CHAMACOS study if they presented for prenatal care at one of six partnering community clinics located throughout the Salinas Valley between October 1999 and October 2000. Women were eligible for the study if they were at least 18 years of age, ≤ 20 weeks gestation, English or Spanish speaking, Medi-Cal eligible, and planning to deliver at the county hospital.

A total of 601 women were enrolled, and after losses due to miscarriage and loss to follow-up, birth weight information was available for 536 children. Women were excluded from the present analysis if they delivered twins (*n* = 5), stillbirths (*n* = 3), or infants born at < 500 g (*n* = 1), as well as women who were diagnosed with pregnancy-induced hypertension (*n* = 15) or diabetes (*n* = 26). Women were also excluded if residential proximity to methyl bromide use could not be assessed for at least 75 days during one trimester of pregnancy (*n* = 44). Participants who were excluded were likely to have relocated at least once during pregnancy (any residential moves: 85% vs. 41%) and tended to be less educated (high school graduate: 15% vs. 22%) and with lower parity (nulliparous: 46% vs. 31%) than participants included in the present analysis. The final sample size was 442 mothers with residential information available for at least one trimester of pregnancy. Written informed consent was provided by all women, and the study was approved by the Committee for the Protection of Human Subjects at the University of California, Berkeley.

*Data collection.* Women were interviewed at the time of enrollment (median, 13 weeks gestation), at the end of the second trimester of pregnancy (median, 26 weeks gestation), and following delivery. All interviews were conducted by bilingual interviewers using structured questionnaires in English or Spanish. Interviews obtained demographic information including maternal age, family income, and number of years lived in the United States, as well as body mass index (BMI) (based on self-reported prepregnancy weight and measured height), and pregnancy history. Information on alcohol, tobacco, drug, and caffeine use was also collected. Women were asked about their occupational status during pregnancy, including work in agriculture. Residential history information was collected by asking participants if they had moved since the last interview and, if so, the dates of all moves.

A home inspection was conducted shortly after enrollment (median, 16 weeks gestation) and when the child was 6 months old. For both visits, latitude and longitude coordinates of the participant’s home were determined using a handheld global positioning system (GPS) unit. Residential mobility during pregnancy was common, with 53% of all participants moving at least once during pregnancy. For this analysis, a woman was included in the sample if her residential location (latitude and longitude coordinates) was known for 75 days or more of at least one trimester of pregnancy (trimester 1: *n* = 338; trimester 2: *n* = 408; trimester 3: *n* = 390). Because we collected latitude and longitude coordinates at two time points, both residential locations were known for women who moved once during pregnancy. For women who moved more than once, residential location was often known for at least one trimester, allowing us to include the woman in the analyses of that trimester and exclude her from the analyses of trimesters where her residence was unknown.

Birth outcome data were collected from medical records and abstracted by a registered nurse. Delivery data included information on pregnancy complications, maternal weight at delivery, infant birth weight (grams), length, head circumference, and gestational age at birth.

**Figure 1:**
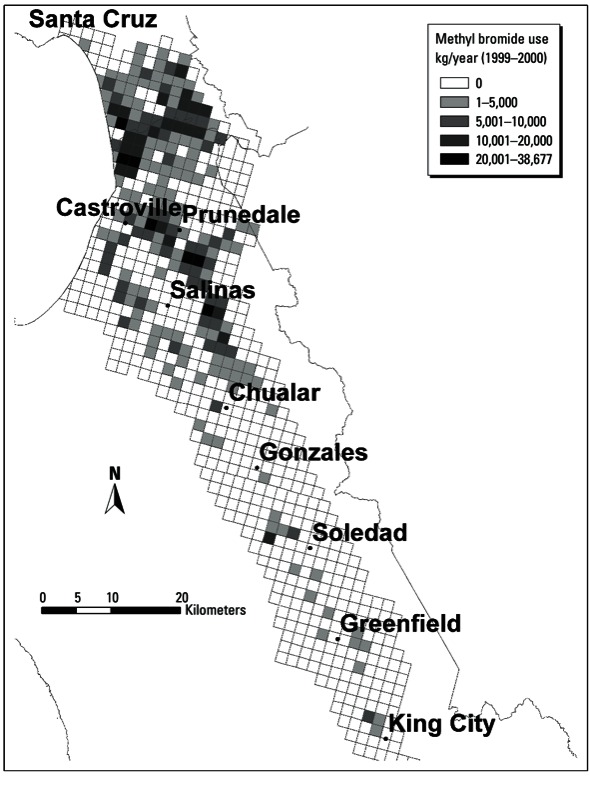
Distribution of methyl bromide use (kg/year) from the California PUR system by section of the PLSS grid in the Salinas Valley, 1999–2000. Data source: California Department of Pesticide Regulation (2012b).

*Geographic-based estimates of methyl bromide use.* We estimated agricultural use of methyl bromide near each woman’s residence using the following variables from the 1999–2000 PUR data: pounds of active ingredient applied, application date, and location. The location of pesticide application is reported for each square mile section (approximately 1.6 km × 1.6 km) defined by the Public Land Survey System (PLSS) (U.S. Bureau of Land Management 2013) (Figure 1). Before analysis, the PUR data were edited to correct for likely outliers with unusually high application rates using previously developed methods (Gunier et al. 2001).

**Figure 2:**
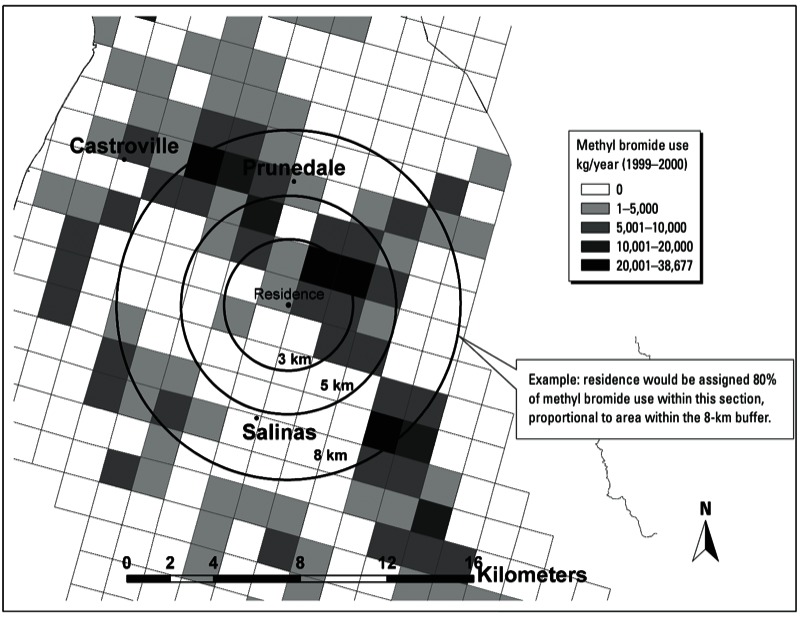
Methods used to determine proximity to methyl bromide use from the California PUR system by section of the PLSS grid illustrated for 3-, 5-, and 8‑km radius buffers around a residence (•). Data source: California Department of Pesticide Regulation (2012b).

For each woman, we calculated the amount of methyl bromide applied within a 5-km radius around her home using a geographic information system (GIS). Detailed descriptions of the equations and methods that were used to calculate pesticide use have been published previously (Gunier et al. 2011; Nuckols et al. 2007). Briefly, we calculated nearby methyl bromide by summing the kilograms applied in all 1.6 km × 1.6 km PLSS sections that fell within 5 km of the maternal residence (Figure 2). For sections that intersected the 5-km buffer, we weighted the amount of methyl bromide applied in that section by the proportion of land area that was included in the buffer. We summed these totals over each day of a trimester interval, yielding an estimate of the total amount of methyl bromide (kilograms) applied within 5 km of the maternal residence during each trimester of pregnancy.

We selected a 5-km buffer distance for this analysis because it best captures the spatial scale most strongly correlated with measured ambient methyl bromide concentrations (Honaganahalli and Seiber 2000; Li et al. 2005; Ross et al. 1996). In particular, Li et al. (2005) investigated the empirical relationship between methyl bromide use and ambient air concentrations in the same agricultural region (Salinas Valley) and time period of interest (2000) as our study using a variety of spatiotemporal scales. They found that parameters for the best-fit model of ambient methyl bromide concentrations at monitoring sites were average methyl bromide use during a 7- to 8-week period over the 7 × 7 mile area surrounding the monitoring site (*R*^2^ = 0.95). Accordingly, we chose a GIS buffer radius of 5 km (3.1 miles) around a residence to adequately capture this 7 × 7 mile area.

We analyzed trimester-specific sums of methyl bromide use in kilograms for each woman as both continuous and categorical variables. We log-transformed (log_10_) continuous methyl bromide variables to reduce the influence of outliers and improve the linear fit of the model. In the categorical analyses, we compared the baseline category of no use (< 1 kg) with moderate use (exposure < median) and high use (exposure ≥ median) groups. To create categories of moderate and high use that were consistent across each trimester, the cut point used was the average of the median values in each of the three trimesters.

*Statistical analysis.* We used linear regression models to estimate associations between methyl bromide usage during each trimester of pregnancy and birth weight, length, head circumference, and length of gestation. All models of birth weight, length, and head circumference were adjusted for gestational age and gestational age squared to estimate the association with fetal growth while adjusting for length of gestation. Potential confounders considered *a priori* were maternal age and week of initiating prenatal care as continuous variables, and parity (nulliparous vs. multiparous), infant sex, mother’s country of birth (born in Mexico vs. other), prepregnancy BMI, family income (at or below vs. above the federal poverty threshold) and mother’s work status during pregnancy (ever paid work vs. no paid work) as categorical variables. Missing covariate values for income level (*n* = 22) were substituted with information from the closest follow-up interview. For women missing height (*n* = 8), prepregnancy BMI was predicted from a regression model that included age, weight, alcohol consumption, and education as predictor variables. We included covariates in the model if their exclusion changed the estimated coefficient for the main effect by > 10% or if they predicted the outcome (*p* < 0.10). Smoking and illicit drug use were not included in the model because few women reported these behaviors and they did not influence main effect estimates. Adjusting for exposure to environmental tobacco smoke and alcohol and caffeine use during pregnancy also did not affect the coefficients of interest.

We conducted sensitivity analyses of alternative buffer distances around the maternal residence, including two smaller distances (1 km and 3 km) and a buffer distance that is slightly larger (8 km) than the 7 × 7 square mile area shown to be optimal for estimating exposure in the Li et al. study (2005). For these analyses, methyl bromide use near the residence was categorized into a dichotomous variable [any use (≥ 1 kg) vs. no use (< 1 kg)]. We also carried out additional sensitivity analyses to investigate the influence of outliers in both the dependent and independent variables.

We conducted all statistical analyses using STATA version 11 statistical software (StataCorp LP, College Station, TX).

## Results

Most of the women in the study were Latina (96%) and born in Mexico (85%) (Table 1). About half were recent immigrants to the United States, having lived in the country for ≤ 5 years. Most mothers lived in families with incomes < 200% of the federal poverty level (96%), and 78% had not graduated from high school. The median age was 25 years (SD = 5), and few women smoked during pregnancy (6%); consumption of ≥ 1 alcoholic drink per week (1%) and illegal drug use (2%) were also low (data not shown). more than half of the sample was either overweight (40%) or obese (22%). Approximately 4% of deliveries were born with low birth weight (< 2,500 g) and 7% were preterm (< 37 weeks gestational age). Characteristics of women in each trimester subsample were similar to those of the total sample [see Supplemental Material, Table S1 (http://dx.doi.org/10.1289/ehp.1205682)].

**Table 1:** Characteristics of study population, CHAMACOS study, Salinas Valley, CA, 1999–2000 (*n* = 442)

Characteristics	*n* (%)
Maternal age (years)
<20	40 (9.1)
20–24	163 (36.9)
25–29	144 (32.6)
30–34	66 (14.9)
≥35	29 (6.6)
Parity
0	139 (31.5)
≥1	303 (68.6)
Race/ethnicity
Latina	426 (96.4)
Non-Latina, white	7 (1.6)
Other	9 (2.0)
Marital status
Married or living as married	356 (80.5)
Unmarried	86 (19.5)
Maternal education
≤6th grade	188 (42.5)
Some high school	158 (35.8)
High school graduate	96 (21.7)
Family income
≤ Poverty	272 (61.5)
Poverty–200%	154 (34.8)
>200%	16 (3.6)
Country of birth
United States	59 (13.4)
Mexico	374 (84.6)
Other	9 (2.0)
Years of residence in the United States
≤5 years	230 (52.0)
6–10 years	99 (22.4)
≥11 years	63 (14.3)
Entire life	50 (11.3)
Work status during pregnancy
Did not work	172 (38.9)
Some field or agricultural work	181 (41.0)
Other work	89 (20.1)
Prepregnancy body mass index (kg/m^2^)
Underweight (<18.5)	2 (0.5)
Normal (18.5–24.9)	170 (38.5)
Overweight (25–29.9)	175 (39.6)
Obese (>30)	95 (21.5)
Smoked during pregnancy
Yes	26 (5.9)
No	416 (94.1)
Any moves during pregnancy
Yes	182 (41.2)
No	260 (58.8)
Low birth weight^*a*^ infant
Yes	17 (3.9)
No	425 (96.2)
Preterm^*b*^ infant
Yes	30 (6.8)
No	412 (93.2)
^***a***^Low birth weight is defined as <2,500g.^***b***^Preterm birth is defined as <37 weeks gestation.	

Table 2 shows the distribution of methyl bromide use for each trimester for the buffer distance of interest (5 km) and the three additional buffer distances used in sensitivity analyses (1, 3, and 8 km). The range of methyl bromide use near residences was similar for trimesters 2 and 3 for each buffer distance, whereas values for trimester 1 were consistently larger due to the presence of 12 outliers (defined as > Q3 + (1.5 × IQR), where Q3 is the value of the 75th percentile (or 3rd quartile) and IQR is the interquartile range). Not surprisingly, the proportion of women living near methyl bromide applications increased as the buffer distance increased. For example, 85% of women lived within 8 km of some amount of methyl bromide use during the first trimester compared to 78%, 60%, and 16% of women for the 5-km, 3-km, and 1-km distances respectively. For the primary buffer distance used in this analysis (5 km), the estimated median amounts of methyl bromide use ranged from 6,010 to 8,201 kg, and the 95th percentile values ranged from 53,685 to 68,893 kg.

**Table 2:** Distribution of methyl bromide use near residences of pregnant women, CHAMACOS study, Salinas Valley, CA, 1999–2000 (*n* = 442).

Group	*n*	No nearby MeBr use (%)	Percentile (kg)			
			25th	50th	75th	95th
1-km radius
Trimester 1	338	83.7	0	0	0	1,669
Trimester 2	408	86.3	0	0	0	766
Trimester 3	390	86.4	0	0	0	615
3-km radius
Trimester 1	338	40.2	0	1,498	13,102	27,718
Trimester 2	408	40.2	0	734	7,379	23,455
Trimester 3	390	42.1	0	291	7,318	23,686
5-km radius
Trimester 1	338	21.6	82	8,201	34,838	68,893
Trimester 2	408	25.5	0	7,419	29,676	53,685
Trimester 3	390	25.4	0	6,010	26,688	59,983
8-km radius
Trimester 1	338	14.5	2,150	16,278	88,413	163,893
Trimester 2	408	18.9	352	11,984	69,200	129,464
Trimester 3	390	18.7	352	10,751	68,333	143,690
MeBr, methyl bromide.						

Table 3 presents estimated associations between methyl bromide use within 5 km of the home as a continuous variable (log_10_) and the four birth outcomes of interest. In minimally adjusted analyses, increases in methyl bromide use near residences in the second trimester were associated with decreases in birth weight, birth length, and head circumference. After adjusting for confounders, associations remained; each 10-fold increase of methyl bromide use in the second trimester was associated with a 21.4-g (95% CI: –43.2, 0.4) decrease in birth weight, a 0.16-cm (95% CI: –0.28, –0.04) decrease in birth length, and a 0.08-cm (95% CI: –0.15, –0.01) decrease in head circumference. Model estimates did not support associations between methyl bromide use in the first or third trimesters and fetal growth. Residential proximity to methyl bromide use was also positively associated with gestational age in the first trimester in both crude and adjusted analysis (adjusted β = 0.13 week; 95% CI: 0.03, 0.22).

**Table 3:** Associations [β (95% CI)] of proximity to methyl bromide use within a 5-km radius (log_10_) during pregnancy with fetal growth parameters and length of gestation, CHAMACOS study, Salinas Valley, CA, 1999–2000 (*n* = 442).

Trimester	Birth weight (g)	*p*-Value	Length (cm)	*p*-Value	Head circumference (cm)	*p*-Value	Gestational age (weeks)	*p*-Value
Minimally adjusted^*a*^
Trimester 1	–5.0 (–30.7, 20.8)	0.71	–0.02 (–0.15, 0.12)	0.82	–0.01 (–0.09, 0.07)	0.85	0.12 (0.02, 0.21)	0.02
Trimester 2	–23.4 (–45.9, –0.9)	0.04	–0.16 (–0.28, –0.03)	0.01	–0.08 (–0.16, –0.01)	0.03	0.00 (–0.09, 0.09)	0.95
Trimester 3	–3.4 (–26.6, 19.8)	0.78	0.01 (–0.11, 0.14)	0.82	–0.06 (–0.13, 0.02)	0.15	0.05 (–0.05, 0.15)	0.30
Adjusted^*b*^
Trimester 1	–11.1 (–36.4, 14.3)	0.39	–0.03 (–0.17, 0.11)	0.69	–0.03 (–0.11, 0.05)	0.43	0.13 (0.03, 0.22)	0.01
Trimester 2	–21.4 (–43.2, 0.4)	0.05	–0.16 (–0.28, –0.04)	0.01	–0.08 (–0.15, –0.01)	0.04	–0.00 (–0.09, 0.09)	1.00
Trimester 3	–0.8 (–23.5, 21.8)	0.94	0.01 (–0.12, 0.14)	0.90	–0.05 (–0.12, 0.03)	0.22	0.03 (–0.06, 0.13)	0.52
^***a***^All models of birth weight, length, and head circumference were adjusted for gestational age and gestational age squared.^***b***^Adjusted for maternal age, parity, prepregnancy BMI, poverty, country of birth, and week of initiating prenatal care.								

Proximity to methyl bromide use was also analyzed as a three-level categorical variable (Table 4). The findings are similar to those from the continuous variable analysis. Using the 5-km buffer, moderate use of methyl bromide (vs. no use) nearby during the first trimester was associated with a 0.70-week increase in gestational age (95% CI: 0.21, 1.18), and high use was associated with a 0.59-week increase (95% CI: 0.12, 1.06). Moderate and high methyl bromide use within 5 km of the home during the second trimester were negatively associated with birth weight (β = –93.1 g; 95% CI: –198.0, 11.7 and β = –113.1 g; 95% CI: –218.1, –8.1, respectively) and birth length (β = –0.62 cm; 95% CI: –1.20, –0.03 and β = –0.85 cm; 95% CI: –1.44, –0.27, respectively), with stronger associations with high versus moderate use. In addition, proximity to moderate or high use in the second trimester was negatively associated with head circumference (β = –0.42 cm; 95% CI: –0.76, –0.08 and β = –0.33 cm; 95% CI: –0.67, 0.01, respectively). There was little evidence of associations between proximity to methyl bromide use during the first and third trimesters and birth weight, length, or head circumference.

**Table 4:** Categorical analysis of associations [β (95% CI)] of proximity to methyl bromide use within a 5-km radius during pregnancy with fetal growth parameters and length of gestation, CHAMACOS study, Salinas Valley, CA, 1999–2000 (*n* = 442).

Group	Range of use (kg)	*n*	Birth weight (g)^*a*^	*p*-Value	Length (cm)^*a*^	*p*-Value	Head circumference (cm)^*a*^	*p*-Value	Gestational age (weeks)^*b*^	*p*-Value
Trimester 1
None	<1	73	Reference		Reference		Reference		Reference	
Moderate^*c*^	1–14,690	121	–13.8 (–141.1, 113.6)	0.83	–0.16 (–0.85, 0.54)	0.66	–0.25 (–0.64, 0.15)	0.21	0.70 (0.21, 1.18)	0.01
High^*d*^	14,690–148,137	144	–27.7 (–149.9, 94.5)	0.66	–0.09 (–0.76, 0.58)	0.80	–0.06 (–0.44, 0.32)	0.75	0.59 (0.12, 1.06)	0.01
Trimester 2
None	<1	104	Reference		Reference		Reference		Reference	
Moderate	1–14,690	152	–93.1 (–198.0, 11.7)	0.08	–0.62 (–1.20, –0.03)	0.04	–0.42 (–0.76, –0.08)	0.02	–0.03 (–0.47, 0.41)	0.89
High	14,690–76,306	152	–113.1 (–218.1, –8.1)	0.04	–0.85 (–1.44, –0.27)	<0.01	–0.33 (–0.67, 0.01)	0.06	–0.06 (–0.51, 0.38)	0.78
Trimester 3
None	<1	99	Reference		Reference		Reference		Reference	
Moderate	1–14,690	155	14.0 (–93.1 121.1)	0.80	–0.04 (–0.64, 0.56)	0.90	–0.27 (–0.62, 0.07)	0.12	0.22 (–0.23, 0.68)	0.33
High	14,690–77,079	136	–23.0 (–133.2, 87.2)	0.68	0.02 (–0.60, 0.64)	0.95	–0.26 (–0.62, 0.09)	0.15	0.18 (–0.29, 0.64)	0.46
^***a***^Adjusted for maternal age, parity, prepregnancy BMI, poverty, country of birth, week of initiating prenatal care, gestational age, and gestational age squared.^***b***^Adjusted for maternal age, parity, prepregnancy BMI, poverty, country of birth, and week of initiating prenatal care.^***c***^Moderate use is defined as less than the median (14,690kg) among participants with any methyl bromide exposure.^***d***^High use is defined as greater than the median (14,690kg) among participants with any methyl bromide exposure.										

Sensitivity analyses were conducted using different buffer distances around the maternal residence. Results for second trimester methyl bromide use (any vs. none) are presented in Table 5 using 1-km, 3-km, 5-km, and 8-km distances. Proximity to methyl bromide use within 1 km and 3 km during the second trimester was negatively associated with body length. Additionally, the 3-km distance yields marginally significant associations with reduced birth weight (*p* = 0.06), reduced head circumference (*p* = 0.08), and increased gestational age (*p* = 0.05). Unlike the smaller buffer distances, the 8-km analysis was more consistent with the 5-km buffer analysis; proximity to methyl bromide use during the second trimester was negatively associated with birth weight, length, and head circumference, and associations were stronger than estimates from the 5-km analysis. Sensitivity analyses with other trimesters yielded few statistically significant associations: In the 3-km analysis there was a significant positive association with first trimester exposure and gestational age [see Supplemental Material, Table S2 (http://dx.doi.org/10.1289/ehp.1205682)].

**Table 5:** Categorical analysis of associations [β (95% CI)] of proximity to any methyl bromide use (vs. none) in the second trimester with fetal growth parameters and length of gestation, CHAMACOS study, Salinas Valley, CA, 1999–2000 (*n* = 442).

Group	*n*	Birth weight (g)^*a*^	*p*-Value	Length (cm)^*a*^	*p*-Value	Head circumference (cm)^*a*^	*p*-Value	Gestational age (weeks)^*b*^	*p*-Value
1-km radius
None	352	Reference		Reference		Reference		Reference	
Any	56	–61.7 (–181.2, 57.8)	0.31	–0.70 (–1.37, –0.04)	0.04	0.07 (–0.32, 0.45)	0.73	–0.23 (–0.73, 0.27)	0.37
3-km radius
None	164	Reference		Reference		Reference		Reference	
Any	244	–81.2 (–165.3, 2.9)	0.06	–0.56 (–1.03, –0.09)	0.02	–0.25 (–0.52, 0.02)	0.08	0.35 (–0.00, 0.70)	0.05
5-km radius
None	104	Reference		Reference		Reference		Reference	
Any	304	–103.1 (–196.5, –9.6)	0.03	–0.73 (–1.25, –0.21)	0.01	–0.37 (–0.68, –0.07)	0.02	–0.05 (–0.44, 0.35)	0.81
8-km radius
None	77	Reference		Reference		Reference		Reference	
Any	331	–150.7 (–254.9, –46.6)	0.01	–1.03 (–1.61, –0.46)	< 0.01	–0.42 (–0.76, –0.08)	0.02	–0.02 (–0.46, 0.42)	0.94
^***a***^Adjusted for maternal age, parity, prepregnancy BMI, poverty, country of birth, week of initiating prenatal care, gestational age, and gestational age squared.^***b***^Adjusted for maternal age, parity, prepregnancy BMI, poverty, country of birth, and week of initiating prenatal care.									

## Discussion

Living within 5 km of methyl bromide use in the second trimester of pregnancy was associated with decreased birth weight, length, and head circumference, despite a positive association between gestational age and residential proximity to methyl bromide use in the first trimester. The estimates for birth weight were only marginally significant (*p* = 0.08) among those women living near moderate methyl bromide use, whereas those for head circumference were marginally significant (*p* = 0.06) among those with high use. Model estimates did not support associations between exposure to methyl bromide during the first and third trimester and fetal growth parameters. Associations with nearby methyl bromide use analyzed as a continuous variable were consistent with those based on categorical exposures: Each 10-fold increase in kilograms of methyl bromide applied within 5 km of the home during the second trimester of pregnancy was associated with a 24.1-g decrease in mean birth weight, a 0.16-cm decrease in mean birth length, and a 0.08-cm decrease in mean head circumference.

The estimated decrease in mean birth weight associated with living near high use during the second trimester (113 g) was about half of the 250-g birth weight decrease generally associated with maternal smoking (Kramer 1987). Nevertheless, the findings suggest that the second trimester may be a critical period for effects of exposure for gestational growth. Findings from studies investigating the effects of maternal smoking (Reeves and Bernstein 2008) and prenatal exposure to wildfires (Holstius et al. 2012) suggest that both the second and third trimesters may be critical periods of growth. In these studies, the authors cite oxidative stress, hypoxia, and suppression of placenta growth hormone as plausible mechanisms.

The results from our sensitivity analyses suggested stronger associations as the buffer distance increased, with negative associations with all three fetal growth parameters for usage within 5-km and 8-km buffer areas. The lack of consistent associations with shorter buffer distances may have been attributable at least partly to exposure misclassification; Li et al. (2005) concluded that using smaller buffers (< 5 km) to determine methyl bromide use underestimates measured outdoor air concentrations. Previous studies using GIS to assess residential exposure to methyl bromide have used a much smaller buffer distance than the present analysis (Cockburn et al. 2011; Reynolds et al. 2004; Rull et al. 2009). These studies selected a buffer distance of 500 m or 0.5 mile (~ 800 m) for all pesticides, but did not use air monitoring data to confirm the validity of these buffer sizes for exposure assessment. In the study by Cockburn et al. (2011), a 500-m buffer was justified based on a previous study that observed high specificity for serum DDE concentrations using a 1-km GIS buffer to estimate organochlorine exposure (Ritz and Costello 2006). Although this shorter buffer distance might be appropriate for assessing exposure to other pesticides, it does not appear to be sufficient for assessing exposure to fumigants, which are likely to drift much further away from the application site (Honaganahalli and Seiber 2000; Li et al. 2005; Woodrow and Krieger 2007). Regulations in California recognize the potential for methyl bromide to drift far from the application site and so restrict methyl bromide use at the township level (6 × 6 square mile sections) to a maximum of 77,848 kg in a calendar month to reduce outdoor air concentrations (California DPR 2010).

The main limitations of this study are that exposure to methyl bromide was based on residential proximity to reported agricultural use, not measured personal air concentrations. Thus time spent in areas with no methyl bromide use or exposure away from home, including at work, was not taken into account. In addition, in our study area and time period, use of chloropicrin, a chemical often used in conjunction with methyl bromide applications, was very highly correlated with methyl bromide use (Spearman correlation = 0.95). As a result, it is not possible to separate associations with methyl bromide from associations with chloropicrin in this study. Use of another pesticide, diazinon, was also correlated with methyl bromide use, but much less strongly (Spearman correlation = 0.44). Future analyses will examine whether diazinon is associated with birth outcomes in this population.

Although we estimated exposure to a specific pesticide that was selected *a priori* based on toxicity and volume of use, other agricultural pesticides could also be associated with fetal growth. However, previous analyses of this cohort did not indicate associations between decreased fetal growth and organophosphate metabolites measured in maternal urine (Eskenazi et al. 2004) or organochlorine concentrations in maternal serum (Fenster et al. 2006).

Methyl bromide use is seasonal, with the most use occurring between August and October. Thus, it is possible that the association we found reflects seasonal patterns in birth weight rather than second-trimester methyl bromide use. However, we did not observe any seasonal patterns in birth weight in this population. Additionally, controlling for birth between February and April (corresponding to a second trimester during August–October) in the multivariable models did not change the results (data not shown).

We did not use a dispersion model incorporating meteorological data to estimate methyl bromide concentrations because dispersion models have not predicted fumigant concentrations as well (*R*^2^ = 0.55–0.82) as the regression model approach (*R*^2^ = 0.95) that we used (Honaganahalli and Seiber 2000; Li et al. 2005). Additionally, location of methyl bromide use was reported to 1-square-mile units. The PUR reporting system does not contain information on applications to specific fields, so it is not possible to ascertain where in the PLSS section the material was applied. However, the potential impact of this misclassification would be small because most PLSS sections fell entirely within the larger buffer distances (e.g., 5 and 8 km) used in our analysis.

Another potential limitation is that women who lived near high use of methyl bromide (vs. moderate or no use) were more likely to live in and around the city of Salinas. It is possible that unmeasured factors related to this group of women might have confounded our results; however, associations remained after controlling for several potential confounders, including maternal education and income. In addition, associations with fetal growth parameters were seen with both moderate and high use of methyl bromide.

Previous studies of perinatal outcomes and agricultural pesticide exposures have relied on the address given in birth certificates and have not incorporated environmental monitoring information in the exposure models (Shirangi et al. 2011). In contrast, for most women, we were able to capture residential location throughout pregnancy, incorporate residential mobility, and estimate exposure separately for each trimester (Canfield et al. 2006). With slightly more than half of our original sample moving at least once during pregnancy, accounting for residential history was important to reduce possible exposure misclassification. Furthermore, residential location was determined using a GPS, which is more accurate than geocoding self-reported addresses (Ward et al. 2005). Another strength of the study was that fetal growth parameters were obtained from medical records, which generally provide more accurate information than birth certificates (Lain et al. 2012). Additionally, because methyl bromide is highly volatile (U.S. EPA 2006), very little exposure to methyl bromide occurs from dietary ingestion or residues brought home on the clothes and skin of occupationally exposed household members; therefore, exposure misclassification related to these sources is not a concern.

The results of this study suggest that living near agricultural applications of methyl bromide may be associated with decreased infant birth weight and other measures of fetal growth. Our finding that residential proximity to methyl bromide use in the first trimester of pregnancy was associated with longer duration of gestation is somewhat puzzling and counter to our expectations. No other epidemiologic studies have specifically examined methyl bromide exposure and fetal growth; therefore, additional studies are needed to confirm the associations we observed. Methyl bromide use in the Salinas Valley declined from 850,000 kg in 2000 to 565,000 kg in 2010 but still represented 29% of soil fumigant use (California DPR 2001a, 2012a). Although critical use exemptions for methyl bromide use are being phased out (U.S. EPA 2012), the lack of fumigant alternatives may prolong its use in the state of California.

## Supplemental Material

(549 KB) PDF
