# Supplementary material for: Residential Proximity to Methyl Bromide Use and Birth Outcomes in an Agricultural Population in California
Source: Environ Health Perspect. 2013 Apr 19;121(6):737–43. doi: 10.1289/ehp.1205682 (PMC3672911; doi:10.1289/ehp.1205682)
Supplement: (549 KB) PDF [file ehp.1205682.s001.pdf]

Environmental Health Perspectives doi:10.1289/ehp.1205682

Supplemental Material
Residential Proximity to Methyl Bromide Use and Birth Outcomes in an Agricultural Population in California

Alison Gemmill, Robert B. Gunier, Asa Bradman, Brenda Eskenazi, Kim G. Harley

<u>Contents</u>

Supplemental Material, Table S1: Characteristics of study population, by trimester exposure……………………………………………………………………………2

Supplemental Material, Table S2: Categorical analysis of associations of proximity to any methyl bromide use (vs. none) in the first and third trimesters with fetal growth parameters and length of gestation………………………………………………...........4

Supplemental Material, Table S1. Characteristics [N (%)] of study population, by trimester exposure, CHAMACOS study, Salinas Valley, CA, 1999-2000 (N=442).

**Characteristics**	**All (N=442)**	**Trimester 1 (N=338)**	**Trimester 2 (N=408)**	**Trimester 3 (N=390)**
Maternal Age				
<20	40 (9.1%)	27 (8.0%)	36 (8.8%)	31 (8.0%)
20 – 24	163 (36.9%)	116 (34.3%)	151 (37.0%)	142 (36.4%)
25 – 29	144 (32.6%)	114 (33.7%)	134 (32.8%)	129 (33.1%)
30 – 34	66 (14.9%)	56 (16.6%)	58 (14.2%)	61 (15.6%)
≥35	29 (6.6%)	25 (7.4%)	29 (7.1%)	27 (6.9%)
Parity				
0	139 (31.5%)	89 (26.3%)	129 (31.6%)	114 (29.2%)
≥1	303 (68.6%)	249 (73.7%)	279 (68.4%)	276 (70.8%)
Race/Ethnicity				
Latina	426 (96.4%)	328 (97.0%)	394 (96.6%)	375 (96.2%)
Non-Latina, White	7 (1.6%)	5 (1.5%)	5 (1.2%)	7 (1.8%)
Other	9 (2.0%)	5 (1.5%)	9 (2.2%)	8 (2.1%)
Marital Status				
Married or living as married	356 (80.5%)	281 (83.1%)	329 (80.6%)	317 (81.3%)
Unmarried	86 (19.5%)	57 (16.9%)	79 (19.4%)	73 (18.7%)
Maternal education				
≤ 6th grade	188 (42.5%)	143 (42.3%)	171 (41.9%)	168 (43.1%)
Some high school	158 (35.8%)	121 (35.8%)	148 (36.3%)	138 (35.4%)
High school graduate	96 (21.7%)	74 (21.9%)	89 (21.8%)	84 (21.5%)
Family income				
At or below poverty	272 (61.5%)	215 (63.6%)	250 (61.3%)	240 (61.5%)
Poverty – 200%	154 (34.8%)	109 (32.3%)	144 (35.3%)	137 (35.1%)
>200%	16 (3.6%)	14 (4.1%)	14 (3.4%)	13 (3.3%)
Country of birth				
United States	59 (13.4%)	41 (12.1%)	54 (13.2%)	54 (13.9%)
Mexico	374 (84.6%)	292 (86.4%)	345 (84.6%)	327 (83.9%)
Other	9 (2.0%)	5 (1.5%)	9 (2.2%)	9 (2.3%)
Years of residence in the U.S.				
≤ 5 years	230 (52.0%)	166 (49.1%)	213 (52.2%)	195 (50.0%)
6 – 10 years	99 (22.4%)	82 (24.3%)	92 (22.6%)	92 (23.6%)
11+ years	63 (14.3%)	56 (16.6%)	58 (14.2%)	58 (14.9%)
Entire life	50 (11.3%)	34 (10.1%)	45 (11.0%)	45 (11.5%)
Work status during pregnancy				
Did not work	172 (38.9%)	127 (37.6%)	156 (38.2%)	153 (39.2%)
Some field or agricultural work	181 (41.0%)	143 (42.3%)	168 (41.2%)	160 (41.0%)
Other work	89 (20.1%)	68 (20.1%)	84 (20.6%)	77 (19.7%)
Prepregnancy body mass index (kg/m^2)				
Underweight (<18.5)	2 (0.5%)	1 (0.3%)	2 (0.5%)	2 (0.5%)

**Characteristics**	**All (N=442)**	**Trimester 1 (N=338)**	**Trimester 2 (N=408)**	**Trimester 3 (N=390)**
Normal (18.5 – 24.9)	170 (38.5%)	122 (36.1%)	158 (38.7%)	142 (36.4%)
Overweight (25 – 29.9)	175 (39.6%)	133 (39.4%)	161 (39.5%)	161 (41.3%)
Obese (>30)	95 (21.5%)	82 (24.3%)	87 (21.3%)	85 (21.8%)
Smoked during pregnancy				
Yes	26 (5.9%)	18 (5.3%)	23 (5.6%)	23 (5.9%)
No	416 (94.1%)	320 (94.7%)	385 (94.4%)	367 (94.1%)
Any moves during pregnancy				
Yes	182 (41.2%)	78 (23.1%)	148 (36.3%)	136 (34.9%)
No	260 (58.8%)	260 (76.9%)	260 (63.7%)	254 (65.1%)
Low birth weight[a] infant				
Yes	17 (3.9%)	12 (3.6%)	16 (3.9%)	17 (4.4%)
No	425 (96.2%)	326 (96.5%)	392 (96.1%)	373 (95.6%)
Preterm[b] infant				
Yes	30 (6.8%)	23 (6.8%)	29 (7.1%)	28 (7.2%)
No	412 (93.2%)	315 (93.2%)	379 (92.9%)	362 (92.8%)

a Low birth weight is defined as <2,500 g

b Preterm birth is defined as <37 weeks' gestation

Supplemental Material, Table S2. Categorical analysis of associations [β (95% CI)] of proximity to any methyl bromide use (vs. none) in the first and third trimesters with fetal growth parameters and length of gestation, CHAMACOS study, Salinas Valley, CA, 1999-2000. (N=442)

Group	N	Birth weight (g)[a]		p-value	Length (cm)[a]		p-value	Head circumference (cm)[a]		p-value	Gestational age[b]		p-value
**Trimester 1**													
1 km: none	283	Ref			Ref			Ref			Ref		
1 km: any	55	-14.4	(-138.5, 109.8)	0.82	0.08	(-0.60, 0.77)	0.81	0.13	(-0.25, 0.52)	0.50	0.19	(-0.29, 0.67)	0.44
3 km: none	136	Ref			Ref			Ref			Ref		
3 km: any	202	-27.7	(-122.1, 66.6)	0.56	0.07	(-0.45, 0.58)	0.80	-0.09	(-0.38, 0.21)	0.56	0.39	(0.03, 0.75)	0.04
5 km: none	73	Ref			Ref			Ref			Ref		
5 km: any	265	-21.5	(-134.6, 91.5)	0.71	-0.12	(-0.74, 0.50)	0.71	-0.15	(-0.50, 0.21)	0.42	0.64	(0.21, 1.07)	<0.01
8 km: none	49	Ref			Ref			Ref			Ref		
8 km: any	289	16.2	(-113.8, 146.2)	0.81	0.26	(-0.45, 0.97)	0.48	-0.11	(-0.52, 0.30)	0.59	0.27	(-0.24, 0.78)	0.30
**Trimester 3**													
1 km: none	337	Ref			Ref			Ref			Ref		
1 km: any	53	-30.6	(-153.8, 92.7)	0.63	0.09	(-0.61, 0.79)	0.80	-0.02	(-0.42, 0.37)	0.91	-0.17	(-0.69, 0.35)	0.53
3 km: none	164	Ref			Ref			Ref			Ref		
3 km: any	226	-5.2	(-90.2, 79.8)	0.90	0.07	(-0.41, 0.55)	0.78	-0.21	(-0.48, 0.06)	0.13	0.23	(-0.12, 0.59)	0.20
5 km: none	99	Ref			Ref			Ref			Ref		
5 km: any	291	-3.2	(-100.2, 93.8)	0.95	-0.01	(-0.56, 0.53)	0.97	-0.27	(-0.58, 0.04)	0.09	0.20	(-0.21, 0.61)	0.33
8 km: none	73	Ref			Ref			Ref			Ref		
8 km: any	317	-22.7	(-130.7, 85.3)	0.68	-0.11	(-0.72, 0.49)	0.72	-0.25	(-0.60, 0.09)	0.15	0.22	(-0.24, 0.68)	0.34

a Adjusted for maternal age, parity, prepregnancy body mass index, poverty, country of birth, week of initiating prenatal care, gestational age, and gestational age squared.

b Adjusted for maternal age, parity, prepregnancy body mass index, poverty, country of birth, and week of initiating prenatal care.
